# Evaluation of ocular surface in children with attention deficit hyperactivity disorder with respect to methylphenidate treatment

**DOI:** 10.5935/0004-2749.2021-0290

**Published:** 2022-09-06

**Authors:** Emre Aydemir, Gözde Aksoy Aydemir, Merve Kalınlı

**Affiliations:** 1 Ophthalmology Department, Adıyaman University Training and Research Hospital, Adıyaman, Turkey; 2 Child and Adolescent Psychiatry Department, Adıyaman University Training and Research Hospital, Adıyaman, Turkey

**Keywords:** Attention deficit disorder with hyperactivity, Methylphenidate/adverse effects, Anterior eye segment, Optical coherence tomography, Dry eye syndromes, Transtorno do deficit de atenção com hiperatividade, Metilfenidato/efeitos adversos, Segmento anterior do olho, Tomografia de coerência óptica, Síndrome do olho seco

## Abstract

**Purpose:**

This study aimed to screen the ocular surface of children with attention deficit hyperactivity disorder and identify the adverse effects of methylphenidate related to dry eye disease.

**Methods:**

This cross-sectional study included children with attention deficit hyperactivity disorder and healthy children (all aged 5-18 years). They were randomized into Group A (without methylphenidate treatment), Group B (with methylphenidate treatment), and Group C (healthy children). Tear film break-up time, Ocular Surface Disease Index questionnaire, tear meniscus height, tear meniscus area, and Schirmer test results were evaluated. Furthermore, symptom severity in attention deficit hyperactivity disorder was assessed by Turgay DSM-IV-based Child and Adolescent Behavioral Disorders Screening and Rating Scale and Conners Parent Rating Scale-48.

**Results:**

Groups A, B, and C consisted of 34, 40, and 60 individuals (n=34, 40, and 60 eyes; age=11.44 ± 2.79, 11.70 ± 2.83, and 11.96 ± 3.63 years, median age=12, 12, and 11.5 years), respectively. Tear film break-up time, Ocular Surface Disease Index, tear meniscus height, tear meniscus area, and Schirmer test results were not significantly different between Groups A and C (p=0.964, 0.336, 0.445, 0.439, and 0.759, respectively). However, Group B showed a significant decrease in tear film break-up time (10.50 ± 3.39 vs. 12.52 ± 2.46 s; p=0.005), tear meniscus height (307.40 ± 5.53 vs. 310.82 ± 7.30 µm; p=0.025), tear meniscus area (0.024 ± 0.0037 vs. 0.026 ± 0.0046 mm^2^; p=0.010) and Schirmer test (12.75 ± 3.96 vs. 15.41 ± 3.75 mm; p=0.004) results compared with Group A.

**Conclusion:**

Compared with healthy children, children with attention deficit hyperactivity disorder showed ocular surface parameters suggestive of dry eye disease despite taking methylphenidate. Thus, they require close ophthalmologic follow-up to prevent sight-threatening dry eye complications.

## Abstract (pt)

**Objetivos:**

Este estudo teve como objetivo examinar a superfície ocular de crianças com transtorno de déficit de atenção com hiperatividade e identificar os efeitos adversos do metilfenidato relacionados à síndrome do olho seco.

**Métodos:**

Este estudo transversal incluiu crianças com transtorno de déficit de atenção e hiperatividade e crianças saudáveis (todas entre 5-18 anos de idade). Elas foram randomizadas no Grupo A (sem tratamento com metilfenidato), Grupo B (com tratamento com metilfenidato) e Grupo C (crianças saudáveis). Foram avaliados o tempo de ruptura do filme lacrimal, questionário sobre Índice de Doenças de Superfície Ocular (IDSO), altura do menisco lacrimal, área do menisco lacrimal e os resultados do teste de Schirmer. Além disso, a gravidade dos sintomas no transtorno de déficit de atenção com hiperatividade foi avaliada usando a *Turgay DSM-IV-based Child and Adolescent Behavioral Disorders Screening and Rating* Scale com base na escala de *Conners Parent Rating Scale-48*.

**Resultados:**

Os Grupos A, B e C consistiram de 34, 40 e 60 indivíduos (n=34, 40 e 60 olhos; idade=11,44 ± 2,79, 11,70 ± 2,83 e 11,96 ± 3,63 anos, idade média=12, 123 e 11,5 anos), respectivamente. O tempo de ruptura do filme lacrimal, o Índice de Doença da Superfície Ocular, as altura do menisco lacrimal, a área do menisco lacrimal e o teste de Schirmer não foram significativamente diferentes entre os Grupo A e C (p=0,964, 0,336, 0,445, 0,439 e 0,759, respectivamente). Entretanto, o Grupo B mostrou uma redução significativa no tempo de ruptura do filme lacrimal (10,50 ± 3,39 vs 12,52 ± 2,46 seg; p=0,005), altura do menisco lacrimal (307,40 ± 5,53 vs 310,82 ± 7,30 µm; p= 0,025), área do menisco lacrimal (0,024 ± 0,0037 vs 0,026 ± 0,0046 mm^2^; p=0,010) e teste de Schirmer (12,75 ± 3,96 vs 15,41 ± 3,75 mm; p=0,004), resultados com0arados com o Grupo A.

**Conclusão:**

Em comparação com crianças saudáveis, crianças com transtorno de déficit de atenção com hiperatividade apresentaram parâmetros de superfície ocular sugestivos de olho seco, apesar do uso de metilfenidato. Assim, elas requerem um acompanhamento oftalmológico próximo para evitar complicações oculares de olho seco que ameaçam a visão.

## INTRODUCTION

Attention deficit hyperactivity disorder (ADHD) is a multifactorial neurodevelopmental disorder that mainly includes genetic and environmental factors^(1)^. Biological markers that can help diagnose ADHD and explain its etiology have been investigated^(2)^. The most acceptable theory is an imbalance in the production of neurotransmitters such as norepinephrine and dopamine in the prefrontal cortex, and medications including methylphenidate hydrochloride (MPH) and amphetamines, which increase dopamine and noradrenaline levels in the synaptic cleft^(3)^.

MPH is a psychostimulant that is used widely for treating ADHD among adolescents and adults^(3)^. Its side effects have been seen in the adult ADHD population but not in children^(4)^. Hence, the potential ocular side effects in children remain poorly studied. Moreover, blink rate (BR) dysfunction may cause damage to the tear film in patients with ADHD^(5,6,7)^.

In this perspective, anterior segment optical coherence tomography (AS-OCT) may be an important tool for studying the dynamics of the lacrimal meniscus and the diagnosis of dry eye disease (DED) in patients with ADHD^(8,9,10)^. Therefore, this study aimed to study the ocular surface of children with ADHD and identify the adverse effects of MPH inducing a secondary DED by evaluating the tear meniscus parameters obtained by AS-OCT, in comparison with healthy children.

Of note, reduction in BR and lacrimal tear production are both harmful to the anterior segment of the ocular surface. Thus, assessing the ocular surface seems crucial in this population.

## METHODS

### Study population and design

Conducted at the ophthalmology department of a tertiary university hospital, this cross-sectional study adhered to the principles of the Declaration of Helsinki. The study protocol was approved by the institutional review board of the Adiyaman University ethics committee (Approval No.: 2021/01-15; Approval date: January 19, 2021). Before enrollment in the study, all of the participants and their parents provided written informed consent.

Caucasian pediatric patients who met the following inclusion criteria were recruited in the patient group. The parents should completely answer the psychological questionnaires for the assessment of ADHD and the degree of their children’s disruptive behavior. They should also complete the Turgay DSM-IV-Based Child and Adolescent Behavioral Disorders Screening and Rating Scale (T-DSM-IV-S)^(11,12)^ and the Conners Parent Rating Scale-48 (CPRS-48)^(13)^. These tests were used to evaluate the participants’ attention deficit, hyperactivity, learning problem, anxiety, conduct disorder, and behavioral changes.

The patient group was further divided into Group A (without MPH treatment [treatment-naïve]) and Group B (with MPH treatment for a minimum of 6 months prior to enrollment into the study). We also added Group C (control), which consisted of patients who attended the ophthalmology department of the institution regularly for eye examinations, did not have any history of ocular surface disease, except for refractive errors, did not have any psychiatric disorders, and did not use any medications.

The Ocular Surface Disease Index (OSDI) questionnaire, tear film break-up time (TF-BUT) analysis, Schirmer test, corneal staining scoring, and AS-OCT were employed in all three groups.

### Patient examination protocol and study measurements

All of the participants completed a comprehensive eye exam, which was conducted by an author (GAA) who was not apprised of the group allocation. The eye exam, which was performed at the ophthalmology department, included the assessment of the ocular motility, best corrected visual acuity (BCVA), fundus photography, and intraocular pressure (IOP). Only participants who had a BCVA of 20/20 or more, a manifested refraction spherical equivalent not greater than ±1 diopter, and an IOP of less than 18 mmHg were included in the study. Conversely, we excluded those who had a primary eye disease (DED, ocular surface disorders, retinal diseases, glaucoma, etc.); ocular inflammation/surgery history; head injury resulting in a loss of consciousness; or immune, neurological, or any other systemic illnesses. Moreover, ocular measurements and tests were conducted on the right eye of each patient between 10 AM and 12 PM, on the same day. In line with the recommendations of the Dry Eye Workshop Group, all tests and measurements included the TF-BUT, corneal staining scoring, and then the Schirmer test^(14)^. The OSDI questionnaire was completed before the ocular tests.

### OSDI

The OSDI is a 12-item questionnaire developed by the Outcome Research Group at Allergan, and it consists of three subscales: 1) ocular symptoms, 2) vision-related function, and 3) environmental triggers^(15)^. As mentioned, we applied this questionnaire to the participants before the ocular test. An OSDI score of 13 or more indicated DED.

### TF-BUT

After applying fluorescein dye solution, we instructed the participants to blink thrice to spread the solution with the tear film. Then, the time between the last blink and the first dark spot that appeared in the cornea was measured. To determine the TF-BUT, we averaged three consecutive measurements. A TF-BUT of less than 10 s indicated DED.

### Corneal staining scoring

After administering a preservative-free solution of 1% fluorescein dye into the conjunctival sac, we examined five corneal areas and then scored the corneal staining; the score ranged from 0 (“absent”) to 3 (“extensive loss of epithelium”)^(16)^.

### Schirmer test

In the Schirmer test, the number of tears produced in 5 min was measured. Briefly, a strip of filter paper was placed between the lateral and middle parts of the lower eyelid. During the test, the patients were asked to look straight forward and blink normally. After 5 min, the amount of wetting on the paper strip was measured in millimeters.

### AS-OCT

The AS-OCT results were read by a masked investigator, and all of the examinations were conducted under the same conditions (temperature: 22°C-25°C, humidity: 30%-50%, time of day: 10 AM-12 PM) in a dimly lit consulting room.

**Figure 1:**
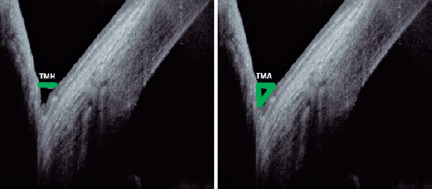
Tear meniscus analysis with anterior segment optical coherence tomography.

The optical coherence tomography (OCT) measurements were conducted using the Spectralis OCT imaging platform (Heidelberg Engineering GmbH, Heidelberg, Germany). Both the lower tear meniscus height (TMH) and the tear meniscus area (TMA) were measured using the Spectralis OCT, with the lens of the anterior segment, in addition to an image-capturing software in the following mode: sclera, high-speed, single-vertical scan. Using the same device, we took scans in the same region, exactly below the corneal vertex, and centered on the inferior cornea and the lower eyelid^(17)^. Furthermore, we used a built-in caliper to measure the TMH in micrometers and the area in square millimeters (Figure 1).

### Statistical analyses

All statistical data were analyzed using IBM SPSS Statistics for Windows 22.0 (IBM Corp., Armonk, NY, USA). We used the chi-square test to compare the categorical values, Kolmogorov-Smirnov test to assess the normal distribution of the variables, and the 2-sample *t*-test to compare the independent variables. Any relationship between the quantitative results was investigated by Pearson correlation analysis. To search for associations between MPH treatment and tear measurements, we used the generalized linear models (GLM). A separate GLM was created for each DED test as a dependent factor. The GLM results were obtained with correlation coefficients (B), lower and upper bounds of 95% Wald confidence interval, and p-values. A p-value of less than 0.05 was considered statistically significant.

## RESULTS

The patient group consisted of 74 participants aged 6-18 years who were referred to the institution’s ophthalmology department by the Child and Adolescent Psychiatry Department. Among these 74 participants, 34 belonged to Group A and 40 belonged to Group B. Meanwhile, Group C was composed of 80 individuals. Table 1 presents the clinical characteristics of these 3 groups. Groups A, B, and C were age-matched (mean age: 11.4 ± 2.79, 11.7 ± 2.83, and 11.9 ± 3.63 [median: 12, 12, and 11.5, range: 6-17, 6-18, and 6-18] years; p=0.742), with female-to-male ratios of 23/11, 27/13, and 38/22, respectively (p=0.876). The mean refractive status was −0.50 ± -0.45 in the patient group and −0.47 ± −0.41 in the control group, showing no significant differences (p=0.73). Furthermore, we noted no significant pathology in the anterior or posterior segment examinations of the groups. The conjunctiva and eyelid margins were examined by slit-lamp biomicroscopy, and none of the groups exhibited meibomian gland disorder, coexistent blepharitis, or fluorescein ocular surface staining. In Group B, the mean duration of MPH treatment was 10.37 ± 2.37 months (min: 6 months, max: 15 months).

**Table 1:** Comparison of the clinical characteristics between the study groups

Variable	ADHD (n=34) Group A	ADHD + MPH (n=40) Group B	Control (n=60) Group C	Statistical analysis			Post hoc comparisons*
				F	df	p-value	
	Mean ± SD	Mean ± SD	Mean ± SD				
Age	11.4 ± 2.79	11.7 ± 2.83	11.9 ± 3.63			0.742¥	
Gender (F/M)	23/11	27/13	38/22			0.876¥	
CPRS							
HA	10.09 ± 3.40	10.85 ± 2.64	5.45 ± 2.14	60.48	2	<0.001	1a > 2, 1b > 2
Learning problems	5.76 ± 1.77	6.13 ± 1.65	4.07 ± 1.19	26.95	2	<0.001	1a > 2, 1b > 2
Anxiety	4.59 ± 2.45	5.60 ± 1.66	3.35 ± 2.37	12.74	2	<0.001	1a > 2, 1b > 2
Psychosomatic	0.85 ± 1.10	0.98 ± 0.89	0.40 ± 0.80	5.49	2	0.005	1b > 2
CD	5.24 ± 2.03	4.55 ± 1.82	3.52 ± 1.01	13.79	2	<0.001	1a > 2, 1b > 2
Parent T-DSM-IV-S							
AD	13.15 ± 2.41	13.58 ± 2.63	7.57± 2.58	85.81	2	<0.001	1a > 2, 1b > 2
HA/I	10.68 ± 3.31	10.43 ± 2.72	5.55 ± 1.84	63.37	2	<0.001	1a > 2, 1b > 2
OD	4.68 ± 2.39	5.70 ± 2.30	3.18 ± 1.01	22.65	2	<0.001	1a > 2, 1b > 2
CD	2.41 ± 1.57	3.03 ± 1.44	1.90 ± 1.03	8.80	2	<0.001	1b > 2

Attention deficit (AD); attention deficit hyperactivity disorder (ADHD), conduct disorder (CD); Conners Parent Rating Scale (CPRS-RS); Turgay DSM IV-based Child and Adolescent Behavioral Disorders Screening and Rating Scale (T-DSM-IV-S); hyperactivity (HA); hyperactivity-impulsivity (HA/I), methylphenidate (MPH); oppositional defiant behavior (OD).

* = Bonferroni test, p<0.05.

¥ = Chi-square test.

The patient group had lower TF-BUT, OSDI, TMH, TMA, and Schirmer test results but had significantly higher corneal staining scores than the control group (11.43 ± 3.15 s, 14.06 ± 2.06, 308.97 ± 6.59 mm, 0.025 ± 0.0043 mm^2^, 13.97 ± 4.07 mm, and 1.29 ± 1.22 [range: 5-18, 7-18, 288-321, 0.016-0.037, 5-23, and 0-4]; p=0.018, 0.033, 0.005, 0.008, 0.016, and 0.001, respectively).

For the subgroup analysis, Group B had significantly lower mean TMH, TMA, TF-BUT, and Schirmer test scores but had significantly higher corneal staining scores than Groups A and C (Table 2).

**Table 2:** Comparison of the study parameters between the subgroups and the control group

	Group A (n=34)	Group B (n=40)	Group C (n=60)	P1*	P2*	P3*
TMH (µm)	310.82 ± 7.30	307.40 ± 5.53	311.68 ± 3.54	**0.025**	**<0.001**	0.445
TMA (mm^2^)	0.026 ± 0.0046	0.024 ± 0.0037	0.027 ± 0.0053	**0.010**	**0.001**	0.439
TF-BUT	12.52 ± 2.46	10.50 ± 3.39	12.55 ± 1.92	**0.005**	**<0.001**	0.964
Schirmer test	15.41 ± 3.75	12.75 ± 3.96	15.66 ± 3.91	**0.004**	**<0.001**	0.759
OSDI score	14.50 ± 1.46	13.70 ± 2.42	15.08 ± 3.33	0.097	**0.026**	0.336
Corneal staining score	0.97 ± 0.83	1.57 ± 1.43	0.68 ± 0.83	**0.033**	**<0.005**	0.112

Tear meniscus height (TMH); tear meniscus area (TMA); tear film break-up time (TF-BUT); Ocular Surface Disease Index (OSDI).

P1= Group A vs. Group B; P2= Group B vs. Group C; P3= Group A vs. Group C.

* = Independent *t* test.

In GLM analysis, significant associations in DED measurements were found in Group B. Significantly lower OSDI scores, TF-BUT, Schirmer test, TMH, and TMA values were associated with MPH treatment (B=-1.383, -2.050, -2.917, -4.283, and -0.003; p=0.011, <0.001, <0.001, <0.001, and <0.001, respectively). In contrast, a higher corneal staining score was significantly associated with MPH treatment (B=0.892, p<0.001). Table 3 presents the GLM results.

**Table 3:** Associations of dry eye disease measurements with MPH treatment (GLM results)

	OSDI	TF-BUT	Schirmer test	TMH	TMA	Corneal staining score
B	−1.383	−2.050	−2.917	−4.283	−0.003	0.892
95% Wald CI	−2.452/−0.315	−3.069/−1.031	−4.456/−1.378	−6.385/−2.182	−0.005/−0.002	0.477/1.306
p	**0.011**	**<0.001**	**<0.001**	**<0.001**	**<0.001**	**<0.001**

Methylphenidate (MPH); generalized linear models (GLM); Ocular Surface Disease Index (OSDI); tear film break-up time (TF-BUT; tear meniscus height (TMH);tear meniscus area (TMA), B; coefficient, CI= confidence interval; The significant p-values are shown in bold.

## DISCUSSION

This study compared the relationship between DED test parameters and ADHD in children. Results showed that children with ADHD had lower TMH, TMA, TF-BUT, Schirmer test, and OSDI scores and higher corneal staining scores than healthy children. Correlations between the OSDI score, TF-BUT, Schirmer test, corneal staining score, TMH, and TMA values, and the duration of MPH treatment were also investigated in patients with ADHD receiving MPH treatment (Group B). Significantly negative correlations were found only between the TMH and MPH treatment duration (r=-0.405, p=0.010), and no correlations with other DED test parameters were observed (p>0.005 for all). However, ADHD severity correlated with the DED test parameters.

This study is the first to draw attention to DED parameters and tear tests in children with ADHD.

Although ADHD neurobiology remains unclear, its main symptoms are generally caused by an imbalance in the noradrenergic and dopaminergic systems. In addition, the suppressing effect of the frontal lobe’s lower center can be either disrupted or not, and the effect of the reticular activating system on the center of attention is reduced^(18)^. Another hypothesis is the dopamine hypothesis in which dopaminergic dysfunction in the brain, which is supported by four main research areas, causes the main symptoms of ADHD^(19)^. MPH is the first line of treatment for ADHD. It is a sympathomimetic amine that has an inhibitory effect on the re-uptake of norepinephrine and dopamine^(20)^.

In a hyperdopaminergic state, spontaneous blinking increases, while reflexive blinking decreases. Conversely, in a hypodopaminergic state, spontaneous blinking decreases, while reflexive blinking increases. However, previous studies on the BRs of individuals with ADHD have reported mixed evidence about these theories. In three studies, which used tasks lasting between 1 and 10 min, any overall differences in the BRs between healthy children (control) and children with ADHD were impossible to determine^(5^, ^6,7)^. One of these studies reported that the BR was lower in children with ADHD than in healthy children during a 5-min period^(21)^, but another study reported no differences in the BR between the same groups^(22)^. Caplan et al.^(5)^ reported specific task effects; for instance, the BR decreased during verbal recall in 21 children with ADHD who did not receive any medical treatment, but it increased while listening in eight children with ADHD who received stimulant medication in comparison with children with normal development. Interestingly, when compared with healthy control participants, children with ADHD did not have a modulated BR across different cognitive tasks, such as listening, verbal recall, and conversation, and the difference in BRs between such tasks was minimal. Therefore, a BR that is intact yet less controlled during task demands suggests the presence of mild suboptimal amounts of norepinephrine and dopamine in the prefrontal cortex. This hypothesis fits quite well with the cognitive and energetic model for ADHD, in which the general structural cognitive processes and energetic state processes (e.g., arousal, activation, and effort), which modulate the structural processes, can be differentiated^(23,24)^.

Both blinking and tears comprise the protective mechanisms of the eye. The normal tear film in the cornea results from the action of blinking, which smoothens out irregularities on the corneal surface in addition to maintaining the symmetric tear-cornea optical interface^(25)^. Given that one of the major functions of spontaneous blinking is the even distribution of the tear film, reduced spontaneous blinking is associated with both the objective and subjective complaints in DED. The impairment of meibomian gland function resulting from decreased BR modulation could possibly lead to tear film abnormalities in patients with ADHD.

Another reason could be the administration of MPH because it also has some ocular side effects^(20)^. According to a case series, corneal edema may develop after undergoing dopaminergic agent treatment^(26)^. In previous experiments, D1 and D2 dopamine receptors were present in the corneas of rabbits but were not uniformly distributed^(27)^. In another animal study, changes occurred in the corneas of rats exposed to MPH at a dose-dependent rate. Initially, the epithelial layer of the cornea was affected; however, as the dose of the medication increased, the effects were observed on both the stroma and endothelial layers. The endothelial cells were disrupted at the level of the junctional complexes^(28)^.

In 2006, Grueb et al. investigated the corneas of human cadavers through immunofluorescence and immunocytochemistry and found that dopaminergic receptors, including D1, were present in the epithelium of the cornea. For confirmation, Western blot analysis was conducted, and the result suggested that dopamine is significant in corneal functions^(29)^. The fact that the same dopaminergic agents do not produce the same toxic effect in everyone is attributed to the sensitivity of the receptor in the endothelial cells of the cornea^(26)^.

In the present study, although a difference was found between patients with ADHD with MPH treatment and the control group, no difference was observed between patients with ADHD without MPH treatment and the control group. As mentioned in the literature, MPH treatment can have a toxic effect on the corneal epithelium and affect the tear test parameters. This finding is supported by the negative correlation between MPH treatment duration and TMH measurements. Patients with ADHD receiving MPH should undergo eye exams at regular intervals. In our study, the OSDI scores were similar, probably because the study group was composed of children, who might have fewer experiences with pain and discomfort; hence, they would be less capable in identifying discomfort caused by a compromised ocular surface^(30)^.

Between the two parameters of the tear meniscus, the TMH values generally had stronger correlations with the ocular tests than the TMA values. Perhaps, the individual anatomical variations in the palpebral structures could have affected the TMA measurements^(31)^.

This study has some limitations that need to be considered when interpreting the results. These limitations include the cross-sectional design of the study, small sample size, wide age range, absence of baseline tests to quantify DED in patients with ADHD receiving MPH treatment, failure to calculate the BR value, and failure to compare the ADHD classification indices with tear measurements.

In conclusion, the tear parameters, TF-BUT, Schirmer test, OSDI scores, and corneal staining scores were in favor of DED in both patients with ADHD with and without MPH treatment. In addition, significant correlations were observed between the Schirmer test, OSDI, corneal staining score, TF-BUT measurements, tear meniscus parameters, and MPH treatment. These findings suggest that the tear meniscus parameters, especially TMH, can reliably be used to evaluate the quantity of tears produced in patients with ADHD, with good repeatability and reproducibility. Baseline examination is recommended before treatment to prevent possible MPH-induced adverse effects on the ocular surface, and close ophthalmological follow-up should be provided after MPH therapy. Additional follow-up longitudinal studies are also necessary to determine if MPH treatment has any effect on the DED parameters and if any differences in these parameters exist between patients with ADHD and healthy controls.

## References

[1] Ercan ES, Polanczyk G, Akyol Ardic U, Yuce D, Karacetın G, Tufan AE (2019). The prevalence of childhood psychopathology in Turkey: A
cross-sectional multicenter nationwide study (EPICPAT-T). Nord J Psychiatry.

[2] Bae S, Kim JT, Han JM, Han DH (2019). Pilot study: an ocular biomarker for diagnosis of attention
deficit hyperactivity disorder. Psychiatry Investig [Internet].

[3] Sharma A, Couture J (2014). A review of the pathophysiology, etiology, and treatment of
attention-deficit hyperactivity disorder (ADHD). Ann Pharmacother.

[4] Agencia Espanola de Medicamentos y Productos Sanitarios (2020). Concerta.

[5] Caplan R, Guthrie D, Komo S (1996). Blink rate in children with attention deficit-hyperactivity
disorder. Biol Psychiatry.

[6] Daugherty T, Quay H, Ramos L (1993). Response perseveration, inhibitory control, and central
dopaminergic activity in childhood behavior disorders. J Genet Psychol.

[7] Jacobsen L, Hommer D, Hong W, Castellanos F, Frazier JA, Giedd JN (1996). Blink rate in childhood-onset schizophrenia: Comparison with
normal and attention-deficit hyperactivity disorder controls. Biol Psychiatry.

[8] Chen F, Shen M, Chen W, Wang J, Li M, Yuan Y (2010). Tear meniscus volume in dry eye after punctual
occlusion. Invest Ophthalmol Vis Sci.

[9] Yuan Y, Wang J, Chen Q, Tao A, Shen M, Shousha MA (2010). Reduced tear meniscus dynamics in dry eye patients with aqueous
tear deficiency. Am J Ophthalmol [Internet].

[10] Ibrahim OM, Dogru M, Takano Y, Satake Y, Wakamatsu TH, Fukagawa K (2010). Application of visante optical coherence tomography tear meniscus
height measurements in the diagnosis of dry eye disease. Ophthalmology.

[11] Turgay A (1994). Disruptive behavior disorders child and adolescent screening and rating
scales for children, adolescents, parents and teachers.

[12] Ercan ES, Amado S, Somer O, Çikoglu S (2001). Development of a test battery for the assessment of attention
deficit hyperactivity disorder. Turk J Child Adolesc Psychiatry.

[13] Derebay Ç, Şener Ş, Derebay İF (1998). Conners parent rating scale adaptation study.

[14] Craig JP, Nelson JD, Azar DT, Belmonte C, Bron AJ, Chauhan SK (2017). TFOS DEWS II report executive summary. Ocul Surf.

[15] Irkec MT, Turkish OSDI Study Group (2007). Reliability and validity of Turkish translation of the ocular
surface disease index (OSDI) in dry eye syndrome. ARVO Annual Meeting Abstract. Invest Ophthalmol Vis Sci.

[16] Lemp MA (1995). Report of the National Eye Institute/Industry Workshop on
Clinical Trials in Dry Eyes. CLAO J.

[17] Arriola-Villalobos P, Fernández-Vigo JI, Díaz-Valle D, Peraza-Nieves JE, Fernández-Pérez C, Benítez-Del-Castillo JM (2015). Assessment of lower tear meniscus measurements obtained with
keratograph and agreement with Fourier-domain optical-coherence
tomography. Br J Ophthalmol.

[18] Spencer T, Biederman J, Mick E (2007). Attention-deficit/hyperactivity disorder: diagnosis, lifespan,
comorbidities, and n, eurobiology. J Pediatr Psychol.

[19] Kirley A, Hawi Z, Daly G, McCarron M, Mullins C, Millar N (2002). Dopaminergic system genes in ADHD: Toward a biological
hypothesis. Neuropsychopharmacology.

[20] Oshika T (1995). Ocular adverse effects of neuropsychiatric agents. Incidence and
management. Drug Saf.

[21] Konrad K, Gauggel S, Schurek J (2003). Catecholamine functioning in children with traumatic brain
injuries and children with attention-deficit/hyperactivity
disorder. Brain Res Cogn Brain Res.

[22] Tantillo M, Kesick C, Hynd G, Dishman RK (2002). The effects of exercise on children with attention-deficit
hyperactivity disorder. Med Sci Sports Exerc.

[23] Sergeant JA (2005). Modeling attention-deficit/hyperactivity disorder: a critical
appraisal of the cognitive-energetic model. Biol Psychiatry.

[24] Sergeant JA, Oosterlaan J, Van der Meere JJ, Quay HC, Hogan AE (1999). Handbook of disruptive behavior disorders.

[25] Huang F, Tseng S, Shih M, Chen FK (2002). Effect of artificial tears on corneal surface regularity,
contrast sensitivity, and glare disability in dry eyes. Ophthalmology.

[26] Mancera N, Wadia HP (2019). Corneal edema associated with systemic dopaminergic
agents. Cornea.

[27] Chang KC, Kim MK, Wee WR, Lee JH (2008). Corneal endothelial dysfunction associated with amantadine
toxicity. Cornea.

[28] Gozil M, Take G, Bahcelioglu M, Tunc E, Oktem H, Caglar G (2008). Dose-dependent ultrastructural changes in rat cornea after oral
methylphenidate administration. Saudi Med J.

[29] Grueb M, Wallenfels-Thilo B, Denk O, Mielke J, Reinthal E, Rohrbach JM (2006). Monoamine receptors in human corneal epithelium and
endothelium. Acta Ophthalmol Scand.

[30] Greiner KL, Walline JJ (2010). Dry eye in pediatric contact lens wearers. Eye Contact Lens.

[31] Chan HH, Zhao Y, Tun TA, Tong L (2015). Repeatability of tear meniscus evaluation using spectral-domain
Cirrus® HD-OCT and time-domain Visante® OCT. Cont Lens Anterior Eye [Internet].

